# Supplementary figures and images for: Mapping quantitative trait loci and predicting candidate genes for leaf angle in maize
Source: PLoS One. 2021 Jan 6;16(1):e0245129. doi: 10.1371/journal.pone.0245129 (PMC7787474; doi:10.1371/journal.pone.0245129)

**Supporting information**

**S1 Fig. The basic situation of the sequencing depth distribution**


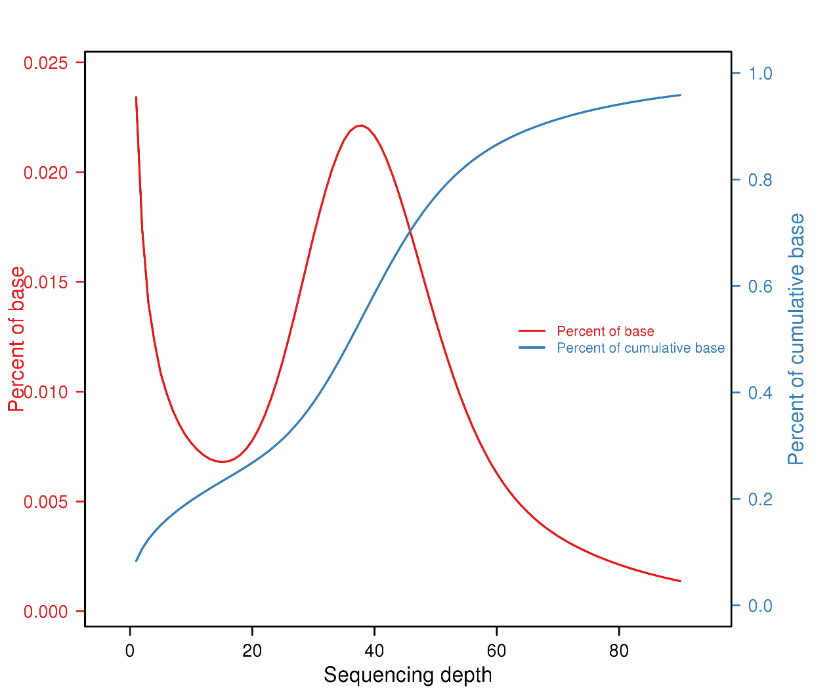

Supplement: S1 Fig — (DOCX) [file pone.0245129.s001.docx]
