# Supplementary material for: Mapping quantitative trait loci and predicting candidate genes for leaf angle in maize
Source: PLoS One. 2021 Jan 6;16(1):e0245129. doi: 10.1371/journal.pone.0245129 (PMC7787474; doi:10.1371/journal.pone.0245129)
Supplement: S2 Fig — The horizontal axis is the chromosomal position, and the vertical axis is the median of read density of the corresponding position on the chromosome (log (2)). There is no significant difference at the 5% level. Error bars indicate the standard deviation of the phenotypic values for each genotype. (DOCX) [file pone.0245129.s002.docx]

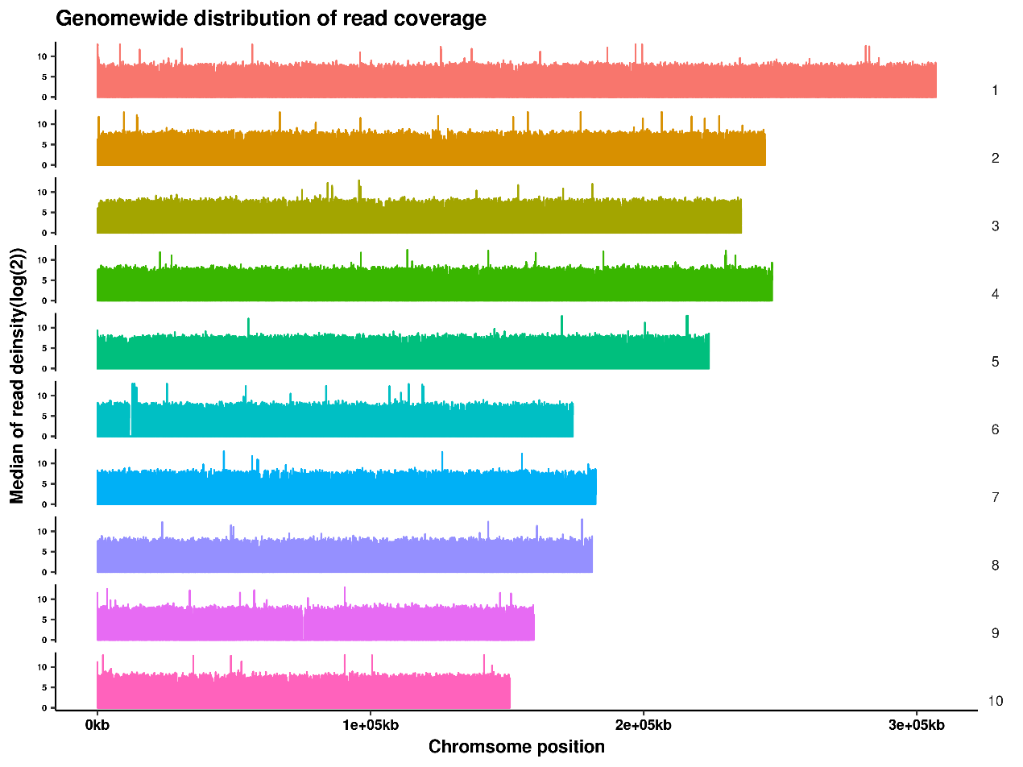
**S2 Fig. Genome wide distribution of read coverage.** The horizontal axis is the chromosomal position, and the vertical axis is the median of read density of the corresponding position on the chromosome (log (2)). There is no significant difference at the 5% level. Error bars indicate the standard deviation of the phenotypic values for each genotype.
