# Supplementary material for: Mapping quantitative trait loci and predicting candidate genes for leaf angle in maize
Source: PLoS One. 2021 Jan 6;16(1):e0245129. doi: 10.1371/journal.pone.0245129 (PMC7787474; doi:10.1371/journal.pone.0245129)
Supplement: S2 Table — (DOCX) [file pone.0245129.s004.docx]

**S2 Table. Whole genome resequencing results of Zheng58.**

| Sample | Raw_Reads | Clean_Reads | Clean_Base | Q20 (%) | Q30 (%) | GC (%) |
| --- | --- | --- | --- | --- | --- | --- |
| Zheng58 | 306686870 | 306237891 | 91690000000 | 97.37 | 92.93 | 45.80 |
