# Supplementary material for: Mapping quantitative trait loci and predicting candidate genes for leaf angle in maize
Source: PLoS One. 2021 Jan 6;16(1):e0245129. doi: 10.1371/journal.pone.0245129 (PMC7787474; doi:10.1371/journal.pone.0245129)
Supplement: Attachment — Submitted filename: Response to Reviewers.docx [file pone.0245129.s007.docx]

Reviewer #1: Please see more comments in the attached PDF. Those marked handwriting comments and suggestions should be addressed to improve the manuscripts. Some figures should be edited and re-upload for publication if accepted.

**Response:** Thanks for your comments. According to your comments and suggestions, we have made some modifications to improve the manuscripts. Some figures have been re-edited and re-upload for publication. Please see the marked-up copy of our manuscript that highlights changes made to the original version. As the manuscript is converted to PDF format, some figures may not be displayed clearly in PDF format. However, each figure in TIFF format in submission system is of high resolution. If the manuscript is accepted, we will provide high-resolution TIFF images to meet the publishing requirements.

Q1 Line 37/76/77 should you change all numbers as letters if they are <10?

**Response:** Thanks for your comments. We have changed all numbers as letters if they are <10. Please see the marked-up copy of our manuscript that highlights changes made to the original version.

Q2 Ten days after pollination (DAP)

**Response:** Thanks for your comments. We have inserted a space after “pollination”. Please see Line 86 in the marked-up copy of our manuscript that highlights changes made to the original version.

Q3 Line85 you only measured 9 data points per line, right? Is it too few?

**Response:** Thanks for your comments. In our work three leaves were measured of each plant. Considering maize plant architecture can be typically evaluated by leaf angle of the three leaves: the first leaf above the primary ear, the primary ear leaf and the first leaf below the primary ear. We randomly choose three out of five plant of each line used for collecting leaf angle data, which can represent the phenotypic value of leaf angle for each line.

Q4 line 151 table1 h2/%?

**Response:** Thanks for your comments. We have deleted “/%” and inserted “(%)”. Please see line 158 table1 in the marked-up copy of our manuscript that highlights changes made to the original version.

Q4 line159-163 Fig 2 this is not a high resolution map. Resolution of Fig 2 is very low, need to redo.

**Response:** Thanks for your comments. We have re-upload a high resolution map for Fig 2.

Q5 Line 183 also the number of SSRs used for mapping? Line183-185 better mention corresponding mbp.

**Response:** Thanks for your comments. In our work, all of the 189 SSR markers with polymorphic between the two parents were used for mapping. In Line183-185, the corresponding Mbp were supplemented. Please see Line191-195 in the marked-up copy of our manuscript that highlights changes made to the original version.

Q6 line 190 Fig 3 use chr or chrom instead of chro for abbreviation.

**Response:** Thanks for your comments. We used chr instead of chro for abbreviation. Please see Fig 3.

Q7 line 200 Theoretically, the leaf angle trait in progenies from selfing HIF155 will be only segregated for qLA01-01.

**Response:** Thanks for your comments. We have changed “selfing” into “selfed”. Please see Line 209 in the marked-up copy of our manuscript that highlights changes made to the original version.

Q8 line 233 explain more?

**Response:** Thanks for your comments. Absolutely, stop gained and start lost, as well as frame-shift mutations often have a greater impact on causing changes in gene function. Thence, these mutations stimulated our further research interest. Please see Line 246-248 in the marked-up copy of our manuscript that highlights changes made to the original version.

Q9 ling316-322 this should be in discussion.

**Response:** Thanks for your comments. These sentences mainly explained the future studies for the qLA02-01 and qLA08-01. There is no particular “Discussion” section in the main text, so we think it's appropriate to put them here.

Reviewer #2: The manuscript mapped 8 major QTLs with F3:4 recombinant inbred lines (RIL) population derived from B73 x Zheng58 for leaf angle and confirmed some of them through a heterogeneous inbred family (HIF) approach. Candidate genes for the qLA02-01 and qLA08-01 regions, with largest contribution effect, were predicted through bioinformatics analysis. The results will benefit to clone the favorable allele for leaf angle or develop the novel maize varieties with ideal plant architecture through marker-assisted selection.
Major issues
Q1 some basic mistakes
L18
Does "biomass" mean “harvest index" here? Because high biomass may not guarantee high grain yield in maize and, in most cases, I believe, it is the grain yield, not biomass, is the major goal for corn production.

**Response:** Thanks for your comments. The grain yield, not biomass, is the major goal for corn production. We deleted "biomass". Please see Line 20 in the marked-up copy of our manuscript that highlights changes made to the original version.

L22
Does the leaf angle refer in particular to that of " the upper leaves of the ear"？

**Response:** Thanks for your comments. In this sentence, the leaf angle refer in particular to the upper leaves of the ear.

L30
11 is the number of all genes that affect leaf angle in maize? or this is only an incomplete statistical number? In my opinion, there are far more known genes involved in leaf angel control in maize. such as CT2 (https://doi. org/10.1371/journal.pgen.1007374)

**Response:** Thanks for your comments. Just as you mentioned, such as CT2, some other genes also affect leaf angle. Here we only listed 11 genes because they were representative genes identified by QTL by mutagenesis and QTL-cloning approach. We modified the sentence. Please see Line 32-33 in the marked-up copy of our manuscript that highlights changes made to the original version.

L173
I guess the auther want to say the B73 allels conribute to the expanded leaf angel with "additive effect". However, it is easiy to lead confusion to change the meaning of a commonly used concept .
**Response:** Thanks for your comments. Here, "additive effect" is easily to lead confusion. We changed "additive effect" to "additive allele effect". Please see Line 180 and Table 2 in the marked-up copy of our manuscript that highlights changes made to the original version.

Q2
L71
Since phenotypes of quantitative traits are easily affected by environment factors, details of the condition during growing seasons, including coordinate, ptotoperiod, temperature and so on should be provided.

**Response:** Thanks for your comments. Environment factors can affect the leaf angle. We supplemented the planting and growing season in the section “Field experiments and statistical analyses”. Please see the Line 78-79 in the marked-up copy of our manuscript that highlights changes made to the original version.

Q3
L69
Were the 165 lines used in the experiment from one F2 ear or 165 F2 ears?

**Response:** Thanks for your comments. The165 lines were selected from one F_2_ ear.

Q4
L75,76
RIL population with 165 lines is small. You may lose some lines during growing in field experiment. Furthermore, phenotypes of quantitative traits are easily affected by QTL X environment effects. However, only two replications were carried out. I need to know how many lines showed good repeatability ? That directly determines the reliability of the data.

**Response:** Thanks for your comments. In this work, more than two hundred lines were planted and 165 lines were survival. Considering that the phenotype of leaf angle was easily affected by environmental conditions, we planted these 165 lines in the same season and the same field in 2014 and 2015. So, the environmental factors affecting leaf angle phenotype were minimized. These 165 lines showed good repeatability in the phenotype of leaf angle and the phenotypic data were used to detected the QTL for leaf angle. Subsequently, we construct the HIF populations to verify the major QTL, each HIF population with at least 120 plants. These plants were also grown in the same season and the same field in 2016. The results verified the authenticity of the QTL mapping results and showed the reliability of the data.

Q5
L230,L272
When do the candidate genes prediction, you may eliminate more irrelevant genes if you take the synonymous mutation into account when select candidate genes in CDS regions.

**Response:** Thanks for your comments. Yes, we eliminated many irrelevant genes by taking the synonymous mutation into account when select candidate genes in CDS. Please see the Line 241and Line285 in the marked-up copy of our manuscript that highlights changes made to the original version.

Q6
Are there any cloned genes that control the leaf angle locate in QTL regions mapped in the manuscript?

**Response:** Thanks for your comments. Yes. Just as shown in Fig 3, some genes has been cloned that control the leaf angle located in the QTL we detected, such as *UPA1*, *lg1* and so on.

Q7
L308
“techniques such as gene editing could be utilized to edit the allele of our candidate genes, which could identify the authentic genes for qLA02-01 and qLA083-1.”
Gene editing do can be utilized to edit the allele of candidate genes. However, in this situation, I believe moderate fine mapping is more operable to eliminate the blindness of the authentic gene identification.

**Response:** Thanks for your comments. We supplemented your suggestion in the text. Please see the line 331 in the marked-up copy of our manuscript that highlights changes made to the original version.

Q8
L83-85
“Three consecutive leaves were measured for each plant, including the first leaf above the primary ear, the primary ear leaf and the first leaf below the primary ear.”
which one of the three leaves was used for data collection of leaf angle? Why?

**Response:** Thanks for your comments. In this work, we measured three leaves and used the mean value for data analysis. Considering maize plant architecture can be typically evaluated by leaf angle of the three leaves: the first leaf above the primary ear, the primary ear leaf and the first leaf below the primary ear. We randomly choose three out of five plant of each line used for collecting leaf angle data.

Q9
“It was noteworthy that all QTL had positive additive effects, suggesting that the B73 parent contributed most alleles for increasing leaf angle (Table 2).”
Why QTL X environment effect was not detected? It is the character of the leaf angle trait, or it resulted from the experiment design because the phenotype was surveyed in the same field, or resulted from the genetic characteristics of selected parents, zheng58 and B73.

**Response:** Thanks for your comments. Considering that the phenotype of leaf angle was easily affected by environmental conditions, we planted these 165 lines in the same season and the same field in 2014 and 2015. These 165 lines showed good repeatability in the phenotype of leaf angle and the phenotypic data were used to detected the QTL for leaf angle. The environmental factors affecting leaf angle phenotype were minimized and the QTL X environment effect was not detected. Subsequently, we construct the HIF populations to verify the major QTL, each HIF population with at least 120 plants. These plants were also grown in the same season and the same field in 2016. The results verified the authenticity of the QTL mapping results and showed that these major QTL are stable and inheritable. In this work, the character of the leaf angle trait resulted from the genetic characteristics of selected parents, zheng58 and B73.

Minor issues
Although, in general, the manuscript was written clearly enough, it still requires extensive editing. eg., There are some grammar mistakes or spelling errors in the manuscript (underlined by red lines), eg. L55, 129,145,260.

**Response:** Thanks for your comments. We have made some modifications for grammar and spelling. Please see the marked-up copy of our manuscript that highlights changes made to the original version.

Many sentences are too wordy and need to be re-organized.
Some figures and tables, such as Fig.5,6, Table 3, are not essentially to contribute to the research results directly could be provided as supplementary data.

**Response:** Thanks for your comments. We have made some modifications for many sentences. Fig.5,6 and Table 3 were removed to supplementary data. Please see S1 Fig., S2 Fig. and S2 Table. in the marked-up copy of our manuscript that highlights changes made to the original version.

L320,
"The cloning and function research of genes in qLA02-01 and qLA08-01" should be more accureate than “The cloning and function research of qLA02-01 and qLA08-01”

**Response:** Thanks for your comments. We made the modification according to your suggestion. Please see Line 333 in the marked-up copy of our manuscript that highlights changes made to the original version.
