# Supplementary material for: Mapping quantitative trait loci and predicting candidate genes for leaf angle in maize
Source: PLoS One. 2021 Jan 6;16(1):e0245129. doi: 10.1371/journal.pone.0245129 (PMC7787474; doi:10.1371/journal.pone.0245129)
Supplement: Attachment — Submitted filename: Response to Reviewer-2.docx [file pone.0245129.s008.docx]

Reviewer #2:

Q1

L22

“The less of leaf angle is, the more upright the upper leaves of the ear are.” I believe “The less of leaf angle is, the more upright the leaves are.” is more accurate. The author is descripting a general concept here, and could not explain it refer in particular to a situation. In case of a particular situation, you may explain it separately. For example, you may say’ The less of leaf angle is, the more upright the leaves are. Upright upper leaves of the ear can maximize photosynthesis efficiency…’here.

**Response:** Thanks for your comments. We have delete “The less of leaf angle is, the more upright the upper leaves of the ear are.” And we have inserted “The less of leaf angle is, the more upright the leaves are.” Please see Line 21 and line 22 in the marked-up copy of our manuscript that highlights changes made to the original version.

L173

Actually, Additive allele effect and Additive effect are with the same meaning to me. The author is still descripting a general concept refer in particular to a situation. To avoid confusion, you may not need to explain Additive effect here, “A positive additive effect value indicates that the B73 allele increases the value of the trait; A negative value indicates that the Zheng58 allele increases the value of the trait.” is clearly enough.

**Response:** Thanks for your comments. Under the table we have deleted “effect of the situation of the Zheng58 allele by the B73 allele”. And just use “A positive additive effect value indicates that the B73 allele increases the value of the trait; A negative value indicates that the Zheng58 allele increases the value of the trait.” as the explanation. Please see the Line178 in the marked-up copy of our manuscript that highlights changes made to the original version.

Q3

L69

I do not understand. If the165 lines were selected from one F2 ear (kernels in the ear are F3 generation), then the single seed descents were from the F3, not F2. Usually, F2 population is a good representative of extensive separation for genes of two parents.

**Response:** Thanks for your comments. Here we did not describe accurately. We have reorganized our description just as “A single seed descent from one F_1_ progeny and then two generations of self-pollination were applied to produce the recombination inbred line population with 165 lines.” Please see the Line69-70 in the marked-up copy of our manuscript that highlights changes made to the original version.

Q4

L75 76

It is better to put the sentence in the manuscript, especially “more than two hundred lines were planted and 165 lines were survival.” to indicate the reliability of data

**Response:** Thanks for your comments. We have inserted the sentence “more than two hundred lines were planted and 165 lines were survival.” Please see Line76-77 in the marked-up copy of our manuscript that highlights changes made to the original version.

Q8

L83-85

Put the sentence” we measured three leaves and used the mean value for data analysis.” in the manuscript in order other researchers could repeat the trial.

**Response:** Thanks for your comments. We have put the sentence “We measured three leaves and used the mean value for data analysis.” Please see the line 84-85 in the marked-up copy of our manuscript that highlights changes made to the original version.

Q9

Although I still believe there should be some QTL X environment effect even the environmental conditions were controlled, if it is not the character of the leaf angle trait, because environmental deviation should exist between years. However, the explication by the author seems acceptable, so put the explication in the manuscript.

**Response:** Thanks for your comments. We have insert the explication into the manuscript. Please see the line218-228 in the marked-up copy of our manuscript that highlights changes made to the original version.
